# Extracellular ATP triggers proteolysis and cytosolic Ca^2+ ^rise in *Plasmodium berghei *and *Plasmodium yoelii *malaria parasites

**DOI:** 10.1186/1475-2875-11-69

**Published:** 2012-03-15

**Authors:** Laura Nogueira Cruz, Maria Aparecida Juliano, Alexandre Budu, Luiz Juliano, Anthony A Holder, Michael J Blackman, Célia RS Garcia

**Affiliations:** 1 Department of Physiology, Instituto de Biociências, Universidade de São Paulo, Rua do Matão, travessa 14, n321, Butantan, 05508-900 São Paulo, SP Brazil; 2 Department of Biophysics, Escola Paulista de Medicina, Universidade Federal de São Paulo, Rua Tres de Maio, 100, Vila Clementino, 04044-020 São Paulo, SP Brazil; 3 Division of Parasitology, MRC National Institute for Medical Research, The Ridgeway, Mill Hill, London, UK NW7 1AA; 4 Department of Parasitology, Instituto de Ciências Biomédicas, Universidade de São Paulo, Av. Prof. Lineu Prestes, 1374 Edifício Biomédicas II, CEP 05508-900 São Paulo, SP Brazil

**Keywords:** ATP, Purinergic receptor, Malaria, *Plasmodium berghei*, *Plasmodium yoelii*, Protease activity, Calcium modulation, Merozoite surface protein 1

## Abstract

**Background:**

*Plasmodium *has a complex cell biology and it is essential to dissect the cell-signalling pathways underlying its survival within the host.

**Methods:**

Using the fluorescence resonance energy transfer (FRET) peptide substrate Abz-AIKFFARQ-EDDnp and Fluo4/AM, the effects of extracellular ATP on triggering proteolysis and Ca^2+ ^signalling in *Plasmodium berghei *and *Plasmodium yoelii *malaria parasites were investigated.

**Results:**

The protease activity was blocked in the presence of the purinergic receptor blockers suramin (50 μM) and PPADS (50 μM) or the extracellular and intracellular calcium chelators EGTA (5 mM) and BAPTA/AM (25, 100, 200 and 500 μM), respectively for *P. yoelii *and *P. berghei*. Addition of ATP (50, 70, 200 and 250 μM) to isolated parasites previously loaded with Fluo4/AM in a Ca^2+^-containing medium led to an increase in cytosolic calcium. This rise was blocked by pre-incubating the parasites with either purinergic antagonists PPADS (50 μM), TNP-ATP (50 μM) or the purinergic blockers KN-62 (10 μM) and Ip5I (10 μM). Incubating *P. berghei *infected cells with KN-62 (200 μM) resulted in a changed profile of merozoite surface protein 1 (MSP1) processing as revealed by western blot assays. Moreover incubating *P. berghei *for 17 h with KN-62 (10 μM) led to an increase in rings forms (82% ± 4, n = 11) and a decrease in trophozoite forms (18% ± 4, n = 11).

**Conclusions:**

The data clearly show that purinergic signalling modulates *P. berghei *protease(s) activity and that MSP1 is one target in this pathway.

## Background

Malaria is one of the most important infectious diseases in the world, responsible for an estimated 655,000 deaths each year [1]. While *Plasmodium *grows and develops inside red blood cells (RBCs), concomitant structural [2] and biochemical changes occurs at the host cell culminating in cell rupture and release of free merozoites[3,4]. It is now well established that *Plasmodium *activates proteases during the blood stages, including during the entry into and exit from its host RBC and the intracellular feeding on haemoglobin [5-9].

As an adaptive evolutionary mechanism, the malaria parasite subverts its host's signalling system to survive and replicate[10-12]. The role of Ca^2+ ^signalling underlying modulation of the *Plasmodium *cell cycle has been extensively investigated including an effect on protease activity[13,14]. For example, some proteases are modulated by intracellular Ca^2+ ^in rodent *Plasmodium *species[15]. Such signalling depends on the maintenance of low cytosolic Ca^2+ ^during its RBC stages[16-26]. However, it is still unknown how a calcium signal is triggered and how particular metabolites derived from the host are central in providing signalling molecules to facilitate parasite growth. The host hormone, melatonin [27] and its derivatives that elicit a rise in cytosolic calcium in *Plasmodium *[17,28-30] also induce proteolysis in *Plasmodium falciparum *and *Plasmodium chabaudi *[31].

Here we have investigated whether other metabolites derived from the host are able to induce proteolysis in *Plasmodium berghei *and *Plasmodium yoelii*. Purines such as adenosine, ADP, ATP and UDP mediate several biological processes[32], being essential in various metabolic cycles and extracellular signalling [33]. The role of ATP in cell signalling is well studied in many eukaryotic cells and includes a wide variety of processes such as secretion, immune responses, mechano-sensory transduction, inflammation, platelet aggregation, cell proliferation, differentiation, and cell migration[34].

ATP is released from RBCs when they are deformed, and this process could also be relevant to malaria parasite invasion since RBCs undergo extensive deformation after the initial merozoite attachment. In addition, it has been reported that following *Plasmodium *infection, the ATP content of RBCs increases [35] and blocking purinergic signalling decreases RBCs invasion by *P. falciparum*[36]. Interestingly, suramin, which inhibits purinergic signalling, has been shown to inhibit merozoite surface protein-1 (MSP1) processing and erythrocyte invasion[37]. In this manner, a role of ATP in Ca^2+ ^signalling and proteolysis to modulate the *Plasmodium *RBC cell cycle is hypothesized.

By using fluorescence resonance energy transfer (FRET) peptides, it was previously shown that Ca^2+ ^modulates protease activation in *P. berghei *and *P. yoelii *parasites[15]. Here, the importance of ATP in modulating proteolysis through Ca^2+ ^pathways in these parasites has been investigated. In addition, the role of ATP in activating proteases or modulating the *P. berghei *cell cycle was studied in the presence of purinergic blocker KN-62. It is shown here that this compound blocks parasite maturation and affects processing of the merozoite surface protein MSP1[38]. Taken together, the present work contributes to the understanding of *P. berghei *biology.

## Materials and methods

### Reagents

Thapsigargin, (phenylmethylsulphonyl fluoride), saponin, probenecid, MOPS (3-(N-morpholino) propanesulfonic acid), EGTA (ethylene glycol-bis (2-aminoethylether)-N,N,N',N tetraacetic acid), adenosine, ATP (adenosine-5'-triphosphate), GTP (guanosine-5'-triphosphate), suramin, PPADS (pyridoxalphosphate-6-azophenyl-2',4'-disulphonic acid), IP5I (diinosine pentaphosphate), TNP-ATP (3'-*O*-(2,4,6-Trinitrophenyl)adenosine-5'-triphosphate tetra(triethylammonium) salt), KN-62 (4-[(2*S*)-2-[(5-isoquinolinylsulfonyl) methylamino]-3-oxo-3-(4-phenyl-1-piperazinyl)propyl] phenyl isoquinolinesulfonic acid ester), Triton X-100 and dihydroethidium were purchased from Sigma- Aldrich (St. Louis, MO). BAPTA/acetoxymethyl ester (AM) and Fluo4/AM were bought from Molecular Probes Inc. (Eugene, OR). The peptide Abz-AIKFFARQ-EDDnp was analytical grade and synthesized according to Hirata, 1994[39-41].

### *Plasmodium berghei (strain NK65) *and *P. yoelii *(strain 17X) parasites

*Plasmodium berghei *and *P. yoelii *were maintained as an asynchronous parasitaemia in mice (Balb/C strain) by transfer every four days. For parasite preparation, filtration of the infected blood through a cellulose column (Whatman CF11) removed leukocytes and platelets. The erythrocytes were then washed twice in PBS (137 mM NaCl, 2.7 mM KCl, 4.3 mM Na_2_HPO_4_, 1.4 mM NaH_2_PO_4_,) by centrifugation at 1,500 *g *for 5 min and lysed in PBS containing 60 μg ml^-1 ^saponin. The membranes were removed by centrifugation (10,000 × *g *for 10 min at 4°C) and further washing of the parasites (1,500 *g *for 5 min) in MOPS buffer (116 mM NaCl, 5.4 mM KCl, 0.8 mM MgSO_4_, 5.5 mM D-glucose, 50 mM MOPS, and 1 mM CaCl_2_, pH 7.2) containing saponin. After erythrocyte lysis, the parasites were maintained in MOPS buffer during the whole experiment.

### Ethical approval

All animal procedures were approved by the São Paulo University Ethics Committee for Animal Experiments (CEEA) according to the Colégio Brasileiro de Experimentação Animal guidelines (COBEA).

### Cell culture of *Plasmodium berghei (strain NK65) *parasites

*Plasmodium berghei *parasites in mice (Balb/C) were transferred to culture at a parasitaemia of 6-10%. The infected RBCs were filtered through a cellulose column (Whatman CF11) as described above and washed twice with RPMI 1640 medium (GIBCO BRL) supplemented with 10% foetal calf serum (FCS). Infected RBCs were then transferred to a culture chamber and kept in suspension by a magnetic stirrer, under an atmosphere of 5% O_2_, 7% CO_2 _and 88% N_2. _The parasites were maintained in culture for 17 h. The stages of intraerythrocytic development were determined by morphology on Giemsa-stained smears.

### Peptide and calcium indicator Fluo4/AM loading

The FRET peptide Abz-AIKFFARQ-EDDnp has a fluorescent group, Abz (*ortho*-aminobenzoic acid), and a quencher group, EDDnp (ethylene diamine-2-4-dinitrophenyl). The peptide is able to access free malaria parasites when the erythrocyte membrane was removed by saponin treatment in MOPS buffer after 1 min incubation. Stock solutions were prepared in DMSO/water (1:1) and concentrations were measured spectrophotometrically using a molar absorption coefficient of 17,300 M^-1 ^cm^-1 ^at 365 nm. For Ca^2+ ^measurements isolated parasites were incubated for 30 min at room temperature with the fluorescent calcium indicator Fluo-4/AM (5 μM) in MOPS buffer containing 1.8 mM probenecid, an inhibitor of organic transport, to minimize indicator extrusion. The cell suspension was then washed three times in MOPS buffer to remove the extracellular dye[17].

### Spectrofluorimetric determinations

Spectrofluorimeric measurements were performed in a Shimadzu RF-5301 PC at 37°C with isolated parasites (10^8 ^cells ml^-1^) incubated with MOPS buffer in a 1 ml cuvette. The fluorescence was measured continuously (acquisition rate: every 0.5 seconds) 1 min after addition of the FRET peptide (10 μM) for 400 seconds. Excitation/emission wavelengths were adjusted to 320/420 nm for Abz and 505/530 nm for Fluo-4 AM. For experiments with purinergic inhibitors parasites were pre-incubated with KN-62, IP5I, TNP-ATP, PPADS or suramin, for 30 min at room temperature. For experiments with the extra or intracellular calcium chelator EGTA (5 mM) or Bapta/AM (25, 100, 200 or 500 μM) parasites were pre-incubated for 5 or 40 min at room temperature, respectively. All incubations were performed before the addition of the FRET peptide.

### Confocal imaging of FRET peptide

Isolated *P. berghei *parasites were resuspended in MOPS buffer and plated onto poly-lysine coated plates (200 μL). After 1 min incubation with the FRET peptide (10 μM), plated cells were taken to a confocal microscope (LSM 510, Zeiss) and observed under a 63× objective (water immersion). Cells were excited with UV laser at 351 nm and 364 nm. To select fluorescence, a 375 nm main dichroic mirror was used. Fluorescence was collected with a 385 long-pass dichroic mirror. Images were taken at 3 seconds intervals and ATP (200 μM) was added to the cells after baseline acquisition. Fluorescence was analysed using LSM 510 Image Examiner (Zeiss).

### Flow cytometry analysis

Using Flow cytometry analysis viability was assessed in *P. yoelii *and *P. berghei *by dihydroethidine (1:200) staining for 20 min at 37°C and analysed by dot plots (side scatter versus fluorescence) of 10^5 ^cells. Dihydroethidine (DHT) was excited with a 488 nm Argon laser and fluorescence emission was collected at 518-605 nm. Parameters subject to adjustment of the FACSCalibur flow cytometer were forward scatter (FSC) (log scale, E-1), SSC (log scale, 269), FL-2 (log scale, 505). For all flow cytometry experiments initial gating was carried out with unstained erythrocytes to account for erythrocyte autofluorescence.

### Immunoblotting

*Plasmodium berghei *infected erythrocytes were incubated in MOPS buffer with Ca^2+ ^(1 mM) and KN-62 (200 μM) or DMSO (0.05%) for 2 h at 37°C. After incubation cells were kept for 2 h at -80°C and subsequently disrupted with lysis buffer (50 mM Tris-HCl pH 8.0, 150 mM NaCl, 1% (v/v) NP-40 and 0.5% (w/v) sodium deoxycholate) including the phosphatase and protease inhibitors (10 μM NaF, 100 μM orthovanadate, 1 μg ml^-1 ^leupeptin, 1 μg ml^-1 ^pepstatin A, 1 μg ml^-1 ^quimostatin, 100 μg ml^-1 ^benzamidine and 1 mM PMSF) for 30 min at 4°C. Samples were quantified by spectrometry and 10 μg of protein was electrophoresed on an 8% polyacrylamide gel and transferred to nitrocellulose. Mouse monoclonal antibody MAb25.1 [42,43] (1:1,000) that specifically binds MSP1[44] was added and incubated overnight at 4°C [45] After washing, blots were incubated for 2 h with secondary HRP-conjugated anti-mouse IgG antibody (1:10,000, GE Healthcare) and binding was detected using enhanced chemiluminescence.

### Statistical analysis

Results are expressed as mean ± SEM of at least three individual experiments. Student's t-test was used for comparisons between two groups, whereas for repeated measures ANOVA was used for comparisons among larger groups. A P value less than 0.05 was considered indicative of a statistically significant difference. GraphPad Prism software (San Diego, CA, USA) was used for all statistical tests.

## Results

Previous studies had shown that extracellular ATP induced a rise of intracellular Ca^2+ ^concentration in *P. falciparum *parasites[36]. Differences in the modulation of proteolysis by Ca^2+ ^among *Plasmodium *species indicated the necessity for new comparative studies to clarify the situation[15,31].

**Figure 1:**
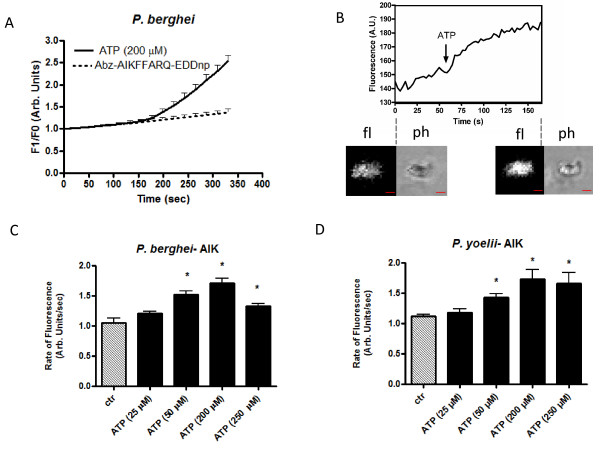
**Extracellular ATP triggers intracellular protease activity in *P. yoelii and P. berghei*. (A) **Graphical representation of resonance energy transfer (FRET) peptide hydrolysis (peptide Abz-AIKFFARQ-EDDnp) in the presence of ATP (200 μM) and the control in isolated (saponin treated) free mixed blood stages of *P. berghei *parasites. **(B) **Fluorescence trace and confocal imaging of Abz-AIKFFARQ-EDDnp hydrolysis. ATP (200 μM) addition is indicated by the arrow. Fluorescence (fl) and phase contrast (ph) before and after ATP addition are below the trace. **(C and D) **Bar graph analyses of peptide hydrolysis at different concentrations of ATP (25, 50, 200 and 250 μM) relative to the control (1.2 ± 0.03, n = 12, *P *= 0.0332; 1.5 ± 0.07, n = 9, *P *= 0.0007; 1.7 ± 0.075, n = 17, *P *< 0.0001 and 1.33 ± 0.04, n = 10; *P *= 0.0048) and (1.17 ± 0.07, n = 5, *P *= 0.425; 1.4 ± 0.06, n = 12, *P *= 0.0039; 1.7 ± 0.15, n = 16, *P *= 0.0267 and 1.7 ± 0.2, n = 8; *P *= 0.0228) for *P. berghei *and *P.yoelii *parasites, respectively. P values were calculated by comparison with the control (ctr) (1.05 ± 0.08, n = 6) and (1.22 ± 0.03, n = 6), respectively. Isolated parasites (10^8 ^cells ml^-1^) were incubated in MOPS buffer with 1 mM calcium in a 1 ml cuvette. The fluorescence was measured continuously (acquisition rate: every 0.5 seconds)1 min after addition of the peptide Abz-AIKFFARQ-EDDnp (10 μM) for 400 seconds.

In the present work, a dose dependent modulation of proteolysis of the FRET peptide substrate Abz-AIKFFARQ-EDDnp induced by ATP (50, 200 and 250 μM) in the presence of extracellular calcium was verified as shown in Figure 1 (panels A, B, C and D). Moreover, when observed under the confocal microscope, the fluorescence of the cleaved substrate Abz-AIKFFARQ-EDDnp clearly colocalizes with *P. berghei *isolated parasites (Figure 1, Panel B). Under these conditions ATP (200 μM) is able to induce proteolysis (Figure 1, Panels C and D). Additional results also showed that adenosine (10 μM) but not GTP (50 μM) induce proteolysis in isolated *P. yoelii *and *P. berghei *parasites (Additional file 1). In these experiments proteolysis was detected by the rate of change in fluorescence caused by hydrolysis of the FRET peptide.

**Figure 2:**
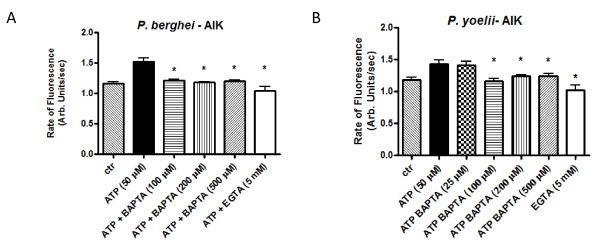
**Calcium is required for FRET activation triggered by ATP in *P. berghei *and *P. yoelii *isolated (saponin treated) free mixed blood stages parasites**. (A and B) The rate of peptide hydrolysis induced by ATP (50 μM) as measured by fluorescence was inhibited after incubation with the extracellular and intracellular calcium chelators: EGTA (5 mM) for 5 min (1.0 ± 0.07, n = 6, *P *= 0.0004) and BAPTA/AM (100, 200 or 500 μM) for 40 min (1.2 ± 0.03, n = 11, *P *= 0.0002; 1.18 ± 0.01, n = 13, *P *< 0.0001; 1.2 ± 0.03, n = 5, *P *= 0.005, respectively) *P *values were calculated by comparison with the ATP (50 μM) data (1.5 ± 0.07, n = 9) in *P. berghei *parasites. *P. yoelii *parasites were also incubated with EGTA (5 mM) (1.02 ± 0.08, n = 7, *P *= 0.0009) and BAPTA/AM (25, 100, 200 or 500 μM) for 40 min (1.4 ± 0.07, n = 7, *P *= 0.823; 1.2 ± 0.03, n = 10, *P *= 0.002; 1.2 ± 0.03, n = 10, *P *= 0.017; 1.2 ± 0.03, n = 10, *P *= 0.022, respectively). P values were calculated by comparison with the ATP (50 μM) data (1.4 ± 0.06, n = 12). Isolated parasites (10^8 ^cells ml^-1^) were incubated in MOPS buffer without CaCl_2. _The fluorescence was measured continuously (acquisition rate - every 0.5 seconds) for 400 seconds.

**Figure 3:**
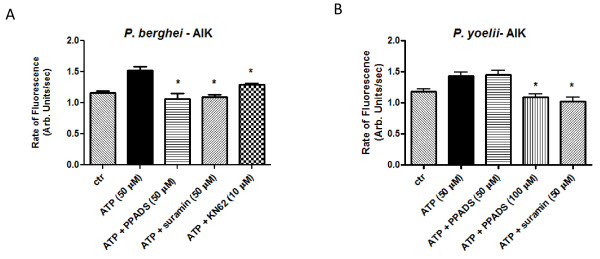
**Inhibitors suggest the presence of purinergic receptor(s) in *P. berghei *(A) and *P. yoelii *(B)**. Rate of peptide hydrolysis induced by ATP (50 μM) as measured by fluorescence after incubation with suramin (50 μM) (1.1 ± 0.04, n = 7, *P *= 0.0002 and 1.0 ± 0.07, n = 7, *P *= 0.0006, respectively), PPADS (50 μM) (1.1 ± 0.08, n = 12, *P *= 0.0008 and 1.4 ± 0.063, n = 6, *P *= 0.791, respectively) or KN-62 (10 μM) (1.3 ± 0.025, n = 13, *P *= 0.001). *P *values were calculated by comparison with the ATP (50 μM) data (1.5 ± 0.067, n = 9 and 1.4 ± 0.062, n = 12, respectively). Effects of ATP on FRET peptide Abz-AIKFFARQ-EDDnp (10 μM) hydrolysis were observed after 30 min incubation of isolated parasites (10^8 ^cells ml^-1^) with the pharmacological agents in MOPS buffer with 1 mM calcium in a 1 ml cuvette._. _The fluorescence was measured continuously (acquisition rate: every 0.5 seconds) for 400 seconds.

To verify whether intracellular and extracellular Ca^2+ ^modulates the proteolytic activity induced by ATP (50 μM), experiments were performed in the presence of BAPTA/AM (25 μM, 100 μM, 200 μM or 500 μM) or EGTA (5 mM), respectively. Figure (2A and 2B) clearly shows that proteolysis was inhibited in the presence of intra- and extracellular Ca^2+ ^chelator indicating the need of the cation in the pathway. Interestingly, it was observed that proteolysis is impaired in the presence of the purinergic inhibitors PPADS (100 or 50 μM) and suramin (50 μM) in *P. berghei *and *P. yoelii *parasites (Figure 3A and 3B, respectively). The P2X (a family of ligand-gated ion channel receptors) inhibitor KN-62 (at 10 μM) also inhibited the proteolytic activity in *P. berghei *parasites (Figure 3A).

**Figure 4:**
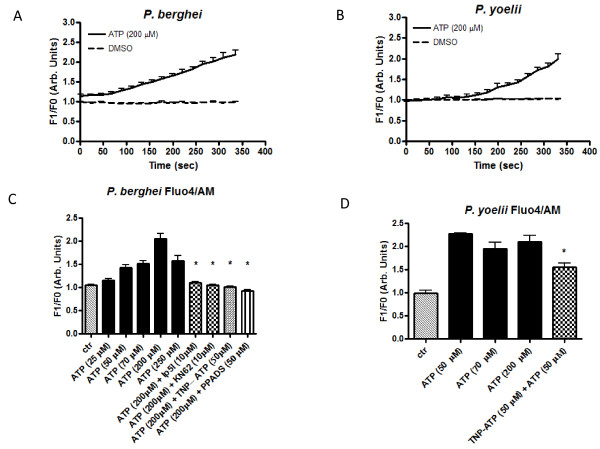
**Dose response effects of ATP on [Ca**^2+^**]_c _are inhibited by purinergic blockers in malarial parasites**. **(A and B) **Representative traces of Fluo4/AM (green fluorescence calcium indicator) changes over time by addition of ATP (200 μM) in *P. berghei *and *P. yoelii*, respectively. **(C and D) **Bar graph analyses of Ca^2+ ^concentration in *P. berghei *and *P. yoelii *Fluo4/AM labelled isolated parasites (10^8 ^cells ml^-1^) after addition of ATP (25, 50, 70, 200 and 250 μM) (1.15 a.u. ± 0.04, n = 8; 1.4 a.u. ± 0.06, n = 14; 1.5 a.u. ± 0.07, n = 11; 1.9 a.u. ± 0.1, n = 15; 1.6 a.u. ± 0.1, n = 11, respectively) in *P. berghei *or ATP (50, 70 and 200 μM) (2.3 a.u. ± 0.02, n = 3; 1.9 a.u. ± 0.1, n = 6; 2.1 a.u. ± 0.14, n = 12), respectively in *P. yoelii*. Mobilization of Ca^2+ ^after treatment with ATP (200 μM) was blocked in the presence of purinoreceptor inhibitors PPADS (50 μM) (0.9 a.u. ± 0.03, n = 9, *P *< 0.0001), TNP-ATP (50 μM) (1.0 a.u. ± 0.01, n = 7, *P *< 0.0001), Ip5I (10 μM) (1.1 a.u. ± 0.02, n = 12, *P *< 0.0001) or KN-62 (10 μM) (1.0 a.u. ± 0.02, n = 9, *P *< 0.0001, respectively) in *P. berghei *and TNP-ATP (50 μM) (1.5 a.u. ± 0.09, n = 9, *P *= 0.001) in *P. yoelii. P *values were calculated by comparison with the ATP (200 or 50 μM) data (2.0 a.u. ± 0.1, n = 12 and 2.3 a.u. ± 0.02, n = 3) in *P. berghei *and *P. yoelii*, respectively. Bar graphs represent means with SEM. The fluorescence was measured continuously (acquisition rate: every 0.5 seconds) for 400 seconds.

Next, whether or not ATP can induce a calcium rise in *P. berghei *and *P. yoelii *using the fluorescent calcium probe Fluo4/AM was investigated. Figure (4A to 4D) shows the typical rise in cytosolic calcium in isolated *P. berghei *and *P. yoelii *after the addition of ATP (50 μM, 70 μM, 200 μM and 250 μM). Addition of the detergent digitonin induced the maximum rise in calcium that was subsequently abolished by addition of the calcium chelator EGTA. Supplementary experiments showed that adenosine (10 μM or 15 μM) is also able to increase cytosolic calcium in *P. berghei *and *P. yoelii *(Additional file 2).

These results indicate that activation of proteolysis by ATP could be triggered by an increase in Ca^2+ ^through purinergic signalling in the rodent malarial parasites. This is better demonstrated by the data in Figures 4C and 4D, where isolated *P. berghei and P. yoelii *parasites that have been loaded with Fluo4/AM were submitted to different pharmacological treatments; the rise in cytosolic calcium was impaired in the presence of P2X blockers TNP-ATP (50 μM), KN-62 (10 μM), Ip5I (10 μM) and PPADS (50 μM).

**Figure 5:**
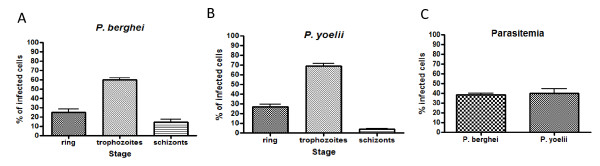
**Distribution of *Plasmodium *stages and parasitemia during experiments**. *P. berghei ***(A) **and *P. yoelii ***(B) **proportion of rings (25.2 ± 3, n = 6 and 27.3 ± 2, n = 6, respectively), trophozoites (60 ± 1.8, n = 6 and 68.8 ± 3, n = 6, respectively) and schizonts (14.7 ± 2.7, n = 6 and 39 ± 0.8, n = 6, respectively). To assess parasitaemia **(C) **(38.4 ± 1.5, n = 6 and 40.1 ± 4.3, n = 6; *P *= 0.718, respectively) in Balb/C mice no less than 1000 erythrocytes were counted on Giemsa-stained smears. Bar graphs represent means with SEM.

The rodent malaria parasites *P. berghei *and *P yoelii *grow as relatively asynchronous populations, therefore, the distribution of intracellular stages during the above experiments (Figures 1, 2, 3, 4) was assessed by morphology using Giemsa-stained smears; the results indicated that the majority of parasites were at the trophozoite stage (Figure 5).

**Figure 6:**
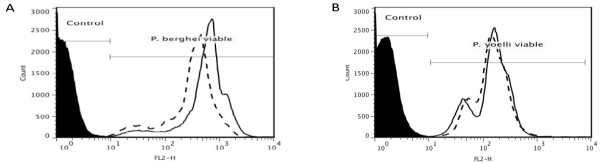
**Flow Cytometry using dihydroethidine staining in *P. berghei *and *P. yoelii *to assess viability**. (A and B) Histogram distribution of fluorescence in non labelled parasites (control), parasites labeled at the beginning (dashed line) and 3 hours later (solid line) in the same buffer. Dihydroethidine is a vital stain taken up by viable parasites, and which then stains nucleic acid. It is the chemically reduced form of DNA intercalating dye ethidium bromide (B-ring reduction). **(C and D) Bar graph analyses of viability in *P. berghei *and *P. yoelii***. The data (mean of three independent experiments) show no statistical difference in DHT fluorescence from the beginning and the end of the experiment (99.8 ± 0.09, n = 3 and 99.8 ± 0.1, n = 3; *P *= 0.823, respectively) and (99.5 ± 0.2, n = 3 and 99.8 ± 0.04, n = 3; *P *= 0.145, respectively).

Parasite viability was verified by flow cytometry analyses using DHT staining. Figure (6A and 6B) shows that both *P. berghei and P. yoelii *parasites were viable at the beginning and end (3 h later) of the spectrofluorimetric assays (99.8% ± 0.04 and 99.5% ± 0.15, n = 3, *P *= 0.144; 99.8% ± 0.1 and 99.7% ± 0.09, n = 3, *P *= 0.822, respectively) indicating ideal experimental conditions for the parasites.

**Figure 7:**
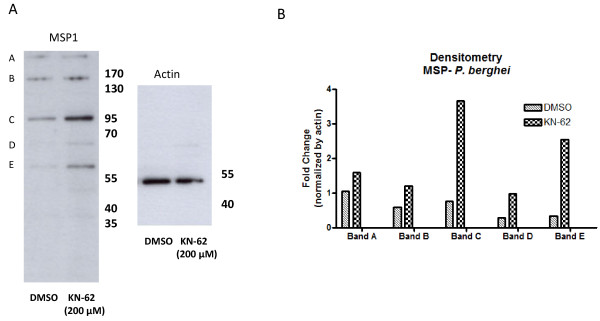
**MSP1 protein expression is increased in KN-62 treated *P. berghei *iRBC. (A) **Representative western blot comparing MSP1 protein and its fragments in control and KN-62 (200 μM) treated *P. berghei *iRBC showing increased expression levels after treatment. Each lane is loaded with 10 μg total cell lysate prepared from freshly harvested parasites. Blots were probed with monoclonal antibody mAb25.1 (1:1000); Anti-actin antibody (1:5000) was used as a control for protein loading. **(B) **Densitometric analysis of blot showed that MSP expression is increased, particularly in bands C and E.

The ability of KN-62 to interfere with the processing of MSP1 in *P. berghei*-infected cells was investigated next. Western blot analyses showed that there is an increased amount of MSP1 and its proteolytic fragments in parasites treated with KN-62 (200 μM) for 2 h (Figure 7A and 7B). MAb25.1 reacts with an epitope in the N-terminal region of MSP1, therefore, band A is the full length precursor (~230 kDa), band C is the N-terminal fragment corresponding to the 83 kDa fragment of *P. falciparum *MSP1, Bands D and E are subfragments of this N-terminal fragment and B is an intermediate fragment containing all of the ~95 kDa fragment C together with other downstream sequences [42-44].

**Figure 8:**
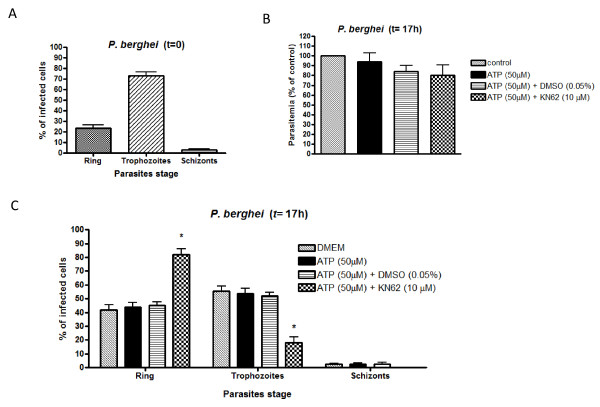
**Effects of purinergic inhibitor KN-62 on *P. berghei *erythrocyte invasion (A)**. *Plasmodium berghei *intraerythrocytic stages (ring, trophozoite or schizont) were assessed during an *in vitro *assay at time zero (beginning) (22.53 ± 2.93, n = 4; 73.21 ± 3.3, n = 4 and 3.26 ± 0.69, n = 4, respectively). **(B) **Culture of asynchronous *P. berghei *parasites were incubated for 17 h in the presence of DMEM (control), ATP (50 μM), ATP (50 μM) with DMSO (0.05%) or ATP (50 μM) with KN-62 (10 μM) and the parasitaemia calculated as a percentage of control (100, n = 4; 93.8 ± 9.3, n = 4; 84 ± 6.1, n = 7 and 80.2 ± 10.4, n = 9, respectively). Note no difference in erythrocyte invasion among the treatments (*P *> 0.05). Distribution of *P. berghei *intraerythrocytic stages in the presence of KN-62 **(C)**. *P. berghei *intraerytrocytic stages: ring (41.9 ± 3.7, n = 9; 43.8 ± 3.6, n = 8; 45.2 ± 2.3, n = 9; 82 ± 4, n = 11; respectively), trophozoite (55.5 ± 3.8, n = 9; 53.7 ± 3.8, n = 8; 52.2 ± 2.5, n = 9; 18 ± 4, n = 11, respectively) or schizont (2.6 ± 0.4, n = 9; 2.5 ± 1, n = 8; 2.6 ± 1.1, n = 9; 0.00 ± 0.00, n = 11; respectively) were assessed after 17 h in the *in vitro *assay. Bars represents the number of rings, trophozoites and schizonts (average), expressed as a percentage of control ± S.E.M. Data were compared by one-way ANOVA and by the Newman-Keuls test. *Statistical significance with respect to control values *P *< 0.001.

The effects of purinoreceptor inhibitor KN-62 on the *P. berghei *cell cycle were verified by maintaining parasites with a similar distribution of development stages (Figure 8A) for 17 h in the presence of KN-62 (10 μM). The data showed a considerable drop in the number of trophozoites (18.01% ± 4.08, n = 11) compared with the number of ring stages (81.99% ± 4.07, n = 11) thus highlighting the relevance of purinoceptors in the parasite developmental cycle (Figure 8C).

## Discussion

ATP is a ubiquitous signalling molecule that recognizes purinergic membrane receptors and modulates several processes studied in a myriad of organisms including slime mould, yeast lizards and mammals (for reviews see [28,46-51]). Purinergic receptors are divided into two classes namely P1 and P2 (adenosine and ATP/ADP, respectively). The P2 receptor includes two types: P2X (family of ligand-gated ion channel receptors) and P2Y (a family of GPCR) [52]. Despite the presence of purinergic signalling mechanisms in invertebrates and lower eukaryotes the evolutionary distance from parasites to human prevents as molecular identification of the receptor from the genome [53-55]. It is noteworthy that in the parasitic helminth *Schistosoma mansoni*, a P2X receptor has been identified in the genome database[56], and using bioinformatic tools Madeira and colleagues identified genes for GPCR-like candidates in the genomes of *P. falciparum, P. berghei, P. yoelii *and *P. chabaudi *[57]. As RBCs do not synthesize their own purines *de novo, Plasmodium *must obtain purine compounds from the extracellular milieu[58]. Endocytosis from the host cell cytosol is thought to be involved in the uptake of nutrients, such as nucleosides, nucleobases and amino acids.

The *P. falciparum *plasma membrane nucleoside transporter, PfENT1 (*P. falciparum *equilibrative nucleoside transporter), has been the subject of extensive study [59,60]. Localized in the plasma membrane [61] its kinetic parameters and substrate specificity have been determined [59,60,62]. The knockout of the PfENT1 gene leads to a reduction in hypoxanthine uptake, and in addition adenosine and inosine transport is affected [63]. The molecular-cellular mechanism by which the parasite obtains extracellular compounds might be related to lipid traffic in the infected RBC [64] and it is well known that the parasite has the ability to create membranous structures in the infected-RBC and new functions such as the anion channel (new permeation pathway)[65-67].

In the present contribution, it was shown that addition of ATP in a dose dependent manner (Figure 1) to *P. berghei *and *P. yoelii *activates intracellular proteolysis. Interestingly, extracellular and intracellular Ca^2+ ^is needed to activate the proteolysis triggered by ATP (Figure 2). It was also found that the purinergic receptor is involved in the ATP signalling pathway to activate the proteases of these rodent malarial parasites since the presence of purinergic antagonists (suramin, PPADS or KN-62) blocked proteolytic activation triggered by ATP in *P. berghei *and *P. yoelii *(Figure 3). Differences in the PPADS concentration needed to block protease activity in *P. berghei *(50 μM) and *P. yoelii *(100 μM) may be due to differences in the total intracellular Ca^2+ ^concentration mobilized by ATP in both specie*s *(Figure 4). This result is in agreement with the presence of a purinergic antagonist (PPADS, TNP-ATP, Ip5I or KN-62) able to inhibit the ability of ATP to induce a rise in calcium in these rodent malarial species (Figure 4). Of interest, the differences in calcium activation of proteolysis between *P. berghei *and *P. yoelii *were reported previously[15].

For the human malaria parasite *P. falciparum*, merozoite invasion and secondary processing of MSP1 is inhibited by suramin[37]. Here it was shown that MSP1 protein expression and processing in *P. berghei *parasites is increased by KN-62 (200 μM) treatment (Figure 7) indicating that MSP1 processing may be a downstream effect of the purinergic signalling pathway of *P. berghei*.

Proteolysis is central to several steps of the *Plasmodium *life cycle including merozoite invasion and egress from RBC, and haemoglobin digestion. In *P. falciparum*, blocking purinergic receptors with either KN-62 or Ip51 prevents parasite invasion of RBCs[36]; interestingly the present work showed that KN-62 modulates the *P. berghei *intracellular cell cycle (Figure 8).

## Conclusion

The data presented here support the concept that *Plasmodium *subverts the host-endocrine system by using extracellular ATP to activate proteolysis to invade or escape from RBCs. The molecular identification of the purinergic receptor in *Plasmodium *as well as the protease (s) involved in these processes represent the basis of new strategies for development of anti-malarial drugs.

## Competing interests

The authors declare that they have no competing interests.

## Authors' contributions

LNC designed the study, collected the data and prepared the first draft. MAJ and LJ synthesized the FRET peptides. AB performed confocal experiment (1B) and wrote the draft related to it. AAH and MJB provided the monoclonal antibody MAb25.1 and analysed the data. CRSG designed the study and prepared the first draft. All authors put forward different ideas, contributed to the interpretation of the data, helped with early drafts and contributed to and agreed the final draft. All authors read and approved the final manuscript

## Supplementary Material

Additional file 1 **Extracellular adenosine but not GTP triggers intracellular protease activity in *P. berghei and P. yoelii *(A and B)**. Bar graph analyses of peptide hydrolysis after GTP (50 μM) (0.99 ± 0.09, n = 5, *P *= 0.63 and 1.06 ± 0.05, n = 9, *P *= 0.365) or adenosine (10 μM) (1.51 ± 0.11, n = 7, *P *= 0.006 and 2.15 ± 0.11, n = 3, *P *< 0.0001) in *P. berghei *and *P. yoelii*, respectively. P values were calculated by comparison with control data (1.05 ± 0.08, n = 6 and 1.12 ± 0.03, n = 6, respectively). Isolated parasites (10^8 ^cells ml^-1^) were incubated in MOPS buffer with 1 mM calcium in a 1 ml cuvette. The fluorescence was measured continuously 1 min after addition of the peptide Abz-AIKFFARQ-EDDnp (10 μM). Click here for file

Additional file 2 **Dose response effects of adenosine on [Ca**^2+^**]_c _rise in *P. yoelii and P. berghei*. (A and B)**. Analyses of Ca^2+ ^concentration in *P. berghei *and *P.yoelii *Fluo4/AM labelled isolated parasites (10^8 ^cells ml^-1^) after addition of adenosine (10 and 15 μM) (1.59 a.u. ± 0.07, n = 18, *P *= 0.007 and 1.5 a.u. ± 0.062, n = 8, *P *= 0.002, respectively) in *P. berghei *or adenosine (10 μM) (1.78 a.u. ± 0.16, n = 8, P = 0.004) in *P. yoelii*. P values were calculated by comparison with control data (1.05 a.u. ± 0.01, n = 3 and 0.99 a.u. ± 0.064, n = 5; respectively). Bar graphs represent means with SEM. Click here for file
