# Supplementary figures and images for: Extracellular ATP triggers proteolysis and cytosolic Ca2+ rise in Plasmodium berghei and Plasmodium yoelii malaria parasites
Source: Malar J. 2012 Mar 15;11:69. doi: 10.1186/1475-2875-11-69 (PMC3358241; doi:10.1186/1475-2875-11-69)

# Supplemental data

S1

A

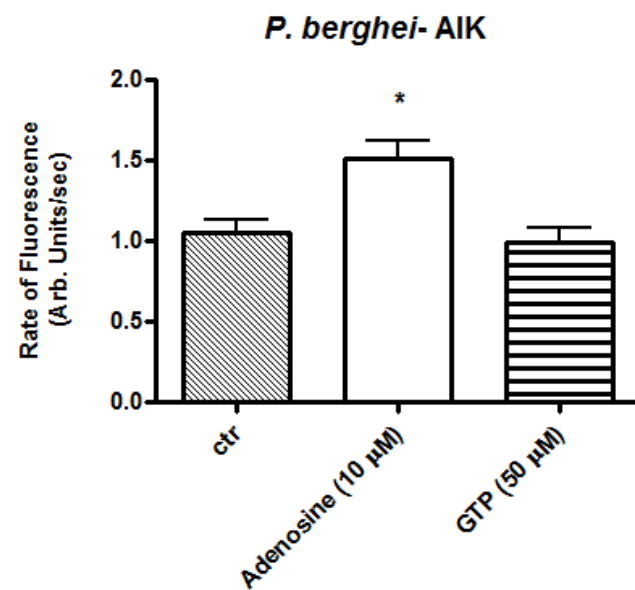

B

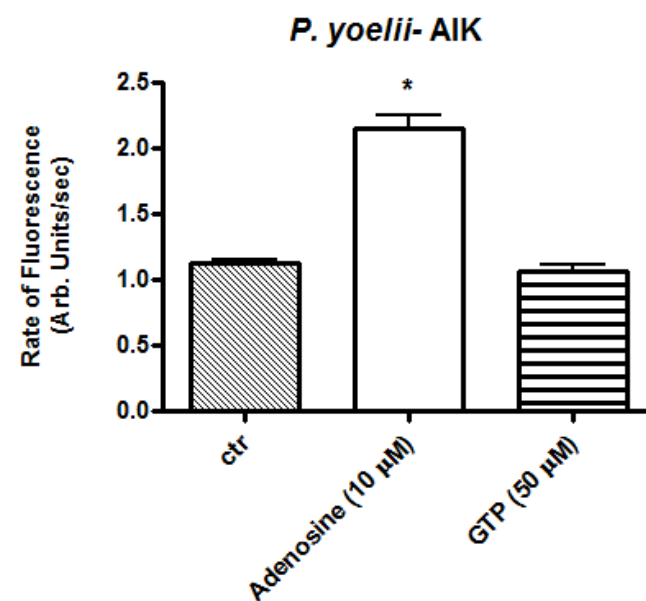

Supplement: Additional file 1 — Extracellular adenosine but not GTP triggers intracellular protease activity in P. berghei and P. yoelii (A and B). Bar graph analyses of peptide hydrolysis after GTP (50 μM) (0.99 ± 0.09, n = 5, P = 0.63 and 1.06 ± 0.05, n = 9, P = 0.365) or adenosine (10 μM) (1.51 ± 0.11, n = 7, P = 0.006 and 2.15 ± 0.11, n = 3, P < 0.0001) in P. berghei and P. yoelii, respectively. P values were calculated by comparison with control data (1.05 ± 0.08, n = 6 and 1.12 ± 0.03, n = 6, respectively). Isolated parasites (108 cells ml-1) were incubated in MOPS buffer with 1 mM calcium in a 1 ml cuvette. The fluorescence was measured continuously 1 min after addition of the peptide Abz-AIKFFARQ-EDDnp (10 μM). [file 1475-2875-11-69-S1.PDF]

# Supplemental data

S2

A

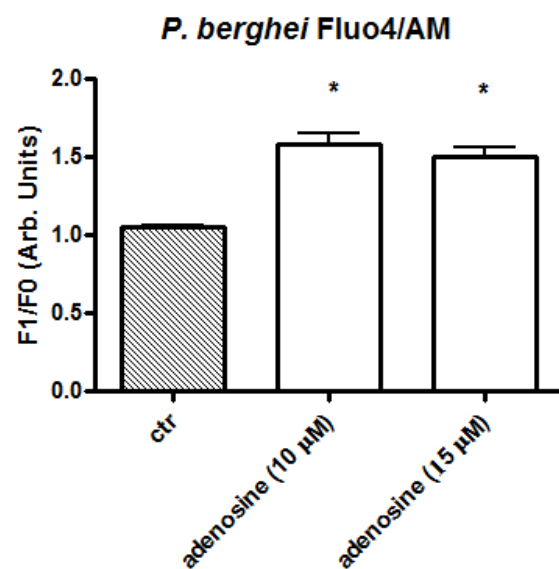

B

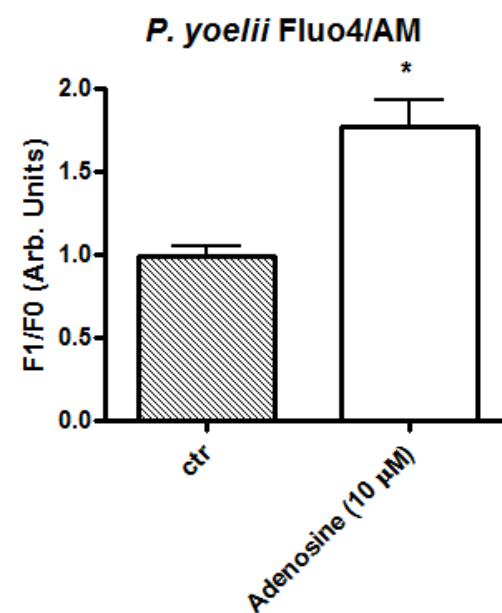

Supplement: Additional file 2 — Dose response effects of adenosine on [Ca2+]c rise in P. yoelii and P. berghei. (A and B). Analyses of Ca2+ concentration in P. berghei and P.yoelii Fluo4/AM labelled isolated parasites (108 cells ml-1) after addition of adenosine (10 and 15 μM) (1.59 a.u. ± 0.07, n = 18, P = 0.007 and 1.5 a.u. ± 0.062, n = 8, P = 0.002, respectively) in P. berghei or adenosine (10 μM) (1.78 a.u. ± 0.16, n = 8, P = 0.004) in P. yoelii. P values were calculated by comparison with control data (1.05 a.u. ± 0.01, n = 3 and 0.99 a.u. ± 0.064, n = 5; respectively). Bar graphs represent means with SEM. [file 1475-2875-11-69-S2.PDF]
